# Towards a MEMS Force Sensor via the Electromagnetic Principle

**DOI:** 10.3390/s23031241

**Published:** 2023-01-21

**Authors:** Rene Hartansky, Martin Mierka, Vladimir Jancarik, Mikulas Bittera, Jan Halgos, Michal Dzuris, Jakub Krchnak, Jaroslav Hricko, Robert Andok

**Affiliations:** 1 Faculty of Electrical Engineering and Information Technology, Institute of Electrical Engineering, Slovak University of Technology, Ilkovicova 3, 812 19 Bratislava, Slovakia; 2 Department of Information Sensor Structures and Technologies, Institute of Informatics, Slovak Academy of Sciences, 845 07 Bratislava, Slovakia

**Keywords:** MEMS, electromagnetic field, voltage controlled oscillator, radiator

## Abstract

Force measurement is a science discipline that experiences significant progress with the introduction of new materials and evaluation methods. Many different sensor types, working on different principles, have been developed and reviewed and have found use in medicine as well as many other industries. New trends and demands require a size reduction and simple applicability, with the use of, for example, micro electromechanical systems (MEMS). For purposes of this study, the initial MEMS body is supplemented by its scaled version. Force measurement in this study works on the force to time-delay conversion principle. A compact compliant mechanical body (CCMB) with an embedded parallel resonant circuit (PRC) acting as a transducer realizes the conversion. Depending on the resonant frequency of the transducer (CCMB or MEMS), we have measured the applied force based on the reverse influence of the transducer on the surrounding EM field. The analysis shows that the transducer’s resonant frequency has a detectable reverse influence on the voltage-controlled oscillator (VCO) DC supply current. The force influencing the transducer is determined by the DC supply current ripple position during the VCO frequency sweep. The study presents the method proposal and mathematical analysis, as well as its function verification by simulation and prototype measurements. The proposed principle was validated on a CCMB prototype capable of measuring forces up to ∼2.5 N at a sampling frequency of ∼23 kHz, while the measured time-delay ranges from 14.5 µs to 27.4 µs.

## 1. Introduction

Mechanical quantity measurement is an essential part of many different industrial or scientific sectors. To prevent damage to the system and any external elements, various acting systems require detailed information about their immediate surroundings and their own components. The necessary information is provided by installed sensors and sensing elements. Taking the importance of sensors into account, the existing and commercially available sensors have been studied in utmost detail, and many different types with very high accuracy, robustness, and overall reliability have been produced. The appearance of robots, autonomous systems, and advanced medical systems requires sensors to be as small as possible and wireless, in addition to the previously mentioned properties. The introduction of new materials, new technologies for precise production, and breakthroughs in evaluation technology allow these new demands to be achieved. Various studies regarding the possible sensor typologies have already been conducted.

The use of dielectric-capacitance-based sensors is particularly popular among these sensors. The working principle of such a sensor is a change in capacitance as a result of an acting force. This sensor type also offers the possibility of detecting the location of an applied force. Authors in [1], with the use of a thin pressure sensor layered upon multi-location capacitance sensors, managed to measure force between 0 N to 10 N and they also managed to locate the area subjected to pressure. Their model needs to be connected via wires, which suffers from various disadvantages. As mentioned before, the use of wiring may prove ineffective when it comes to spatial requirements. The use of wiring may also prove to have difficulties with possible external interference, which may lead to invalid readings.

Other studies focused on eliminating the issues associated with the use of wiring in favor of wireless applications. The use of antennas, particularly patch antennas connected directly to the sensing element, appears to be a popular and effective topic for such studies. A very interesting idea proved to be the connection of the sensing element to the antenna via a micro-strip transmission line, as presented in [2]. The study develops a millimeter-sized sensing element that can measure pressures up to 4 MPa using surface acoustic wave SAW)-sensing technology. The signal transfer is realized by a slotted patch antenna and transmission line. The sensing element is excited by the received electromagnetic wave and changes resonance based on the pressure stressing the element. This approach may yield a wide range of uses by eliminating any type of wire connection to the evaluation or measurement system. While the sensing element itself is small, the antenna and the transmission line are characterized by the operating frequency and substrate properties. The study utilized an operating frequency of 915 MHz, which yielded a transmission line of 150 mm in length and a patch antenna with the largest dimension being 45 mm. The evaluation of the resulting signal requires a highly specialized and expensive measurement system, such as a network analyzer. Some other methods of sensing element resonance and its application in harsh environments are presented in [3,4].

Another interesting approach to wireless sensor technology based on backscattering is described in [5]. The force sensing in the study is based on an investigation of reflected wave phase. The proposed sensor comprises two antennas, a signal trace, a rigid ground plane, and compliant layers. The signal trace is a half-wave transmission line situated above the rigid ground plane. Applied force causes the signal line to create a short with the ground plane, resulting in a phase-shifted reflected wave. The phase shift carries information about the location and magnitude of the applied force. The antenna transmits the reflected wave, with the phase shift carrying information about the applied force. This type of sensor is again restricted by the transmission line–frequency relationship, resulting in large dimensions for lower frequencies. Other possible approaches to wireless information transfer can be found in [6,7,8].

All of the force measurement methods mentioned are valid and interesting, but they usually necessitate the use of a network analyzer to evaluate the data from the measurement setup.

The previous study [9] explored the possibility of wireless transfer of information between the transducer (sensing element) (physical quantity -> electrical quantity) and the electronic part of the sensor. The transducer (composed of CCMB and an embedded PRC) is passive and does not require any galvanic connection to a power source or evaluating electronics. It is powered by an external EM field created by the radiator. Eliminating any kind of wiring between the sensing element and other parts of the sensor offers many advantages and possible future uses in small and confined spaces. In this study, the transducer was placed in a fully closed radiator, generating the EM field, to create the best possible conditions for measurement. For practical uses, the enclosed radiator is not necessary, which allows the transducer to be placed anywhere where there is the possibility to create a known EM field. The previous study proved that applied force on the transducer is seen as a change in the converter resonant frequency, which results in changing the amplitude of the reflected wave. The most significant change occurs at frequencies near the resonant frequency of the transducer and has a detectable reverse influence on the surrounding EM field. As a result, the force applied to the transducer body causes an overshoot in the voltage standing wave ratio (VSWR) at the input of the radiator. The reflected wave was isolated by a directional coupler and evaluated by uP. The reflected wave is a high-frequency signal, and its evaluation requires specialized RF components, such as a directional coupler, and uP with enough computing power and complicated software due to the frequency of measurements up to hundreds of MHz. This significantly complicates the applicability in the commercial sector, and therefore different methods of the overshoot detection had to be developed.

The main goal of this study was to present a new method of transducer EM field influence detection that circumvents the RF components. The following chapters will focus on the transducer principle description, the mathematical analysis of converter (radiator + transducer), synthesis of the equivalent circuit used in simulation, and the simulation proof of the VCO DC supply current change caused by transducer property changes. A newly developed electronic part and measurements utilizing the new method of transducer EM field influence detection are presented as well.

## 2. Operation Principle

**Figure 1:**
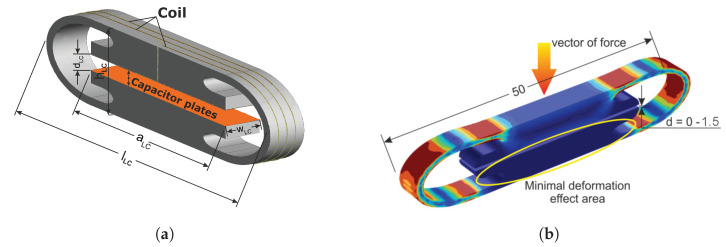
Visualization of the experimental transducer seen in Figure 2 : $l_{LC}$ = 52 mm, $h_{LC}$ = 10.5 mm, $w_{LC}$ = 5.6 mm, $a_{LC}$ = 37 mm, $d_{LC}$ = 1.75 mm (compact compliant mechanical body with inductor and capacitor in parallel). (**a**) Distribution of von Mises stress and (**b**) deformation of the elastic element.

**Figure 2:**
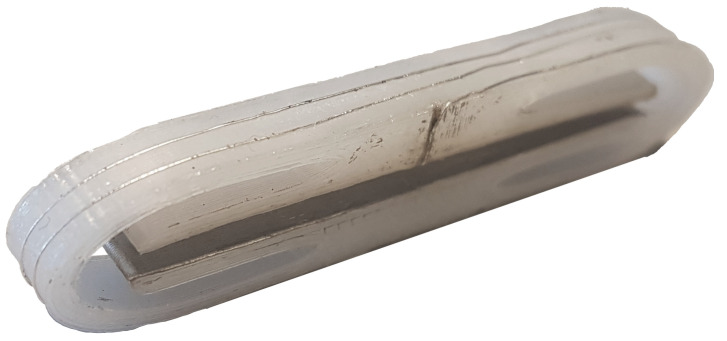
Experimental transducer of a single-component force.

**Figure 3:**
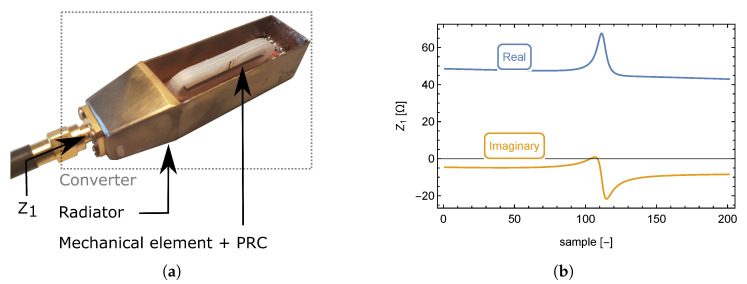
Photograph of a mechanicalelement + PRC, (creating transducer), inside the radiator (creating converter as a whole) (**a**). converter input impedance $Z_{1}$, as function of normalized frequency (see Equation (2)) (**b**).

**Figure 4:**
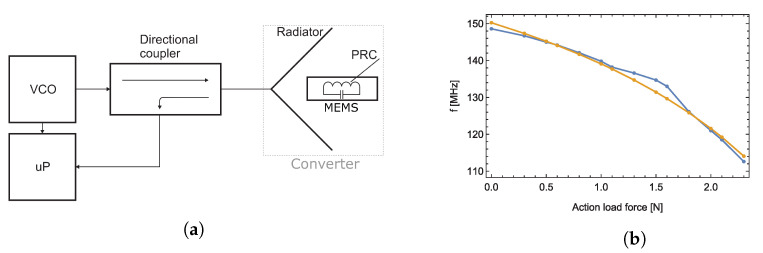
Block diagram of sensor, for our previous measurement and evaluation method (**a**) Transfer characteristic between applied force and the resonant frequency of converter, measured with the use of previously published method (yellow—theory, blue—measurement) (**b**) [9].

**Figure 5:**
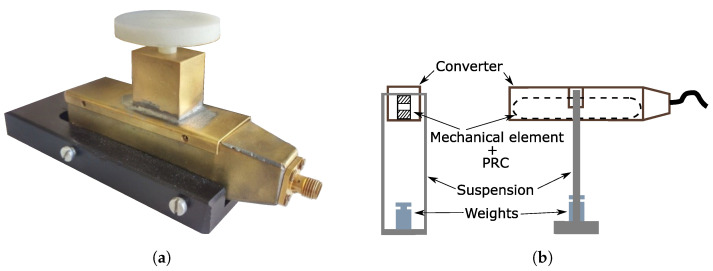
Loading structure with TEMcell (**a**). Suspended version of loading structure with TEMcell (**b**).

Measurement of force with the use of passive transducers is usually accomplished by an LC resonant circuit with a variable capacitance. The variable capacitance is achieved by creating a compliant mechanical body that slightly changes its shape when subjected to force, as demonstrated in Figure 1b. The CCMB design allows the creation of capacitor plates in the middle of the compliant body, while the threads of an inductor are embedded in the exterior portion of the compliant body, creating a parallel resonant circuit (PRC) as can be seen in Figure 1a. Different CCMB designs and materials were tested and are presented in [9]. The mechanical body was cut out of Teflon using a water jet, according to [10] with dimensions of 60×15×6 mm. The PRC inductor comprises a silver wire, and the capacitor was constructed by applying a silver layer to parallel plates of a CCMB. The final compliant mechanical body design of the experimental transducer is shown in Figure 2. During the measurement, the transducer was placed inside the radiator as shown in Figure 3a and the radiator was connected to the electronic part of sensor according to the block diagram seen in Figure 4a. The acting force was created by adding individual weights to the stressing mechanism presented in Figure 5a and applied to the middle section of the CCMB as shown in Figure 1b. The acting force is typically measured with an external driving antenna, and S-parameters are measured with a network analyzer. To avoid the need for such large and expensive laboratory equipment, we have tested and proven an evaluation method based on measuring the reflected wave from the radiator (the generator of the EM field), as shown in Figure 4a [9]. The reflected wave was processed by a microcomputer board (instead of a network analyzer), and the overshoot in the voltage standing wave ratio (VSWR) was used to determine the resonant frequency of the transducer. The transfer characteristic between applied force and transducer resonant frequency measured with this method is depicted in Figure 4b [11].

While this method of detecting the resonant frequency of the transducer does not require any laboratory instruments, it still uses many high-frequency components, which complicates the design of such an evaluating circuit. Due to frequency measurements up to hundreds of MHz (frequency requirements can be in the GHz range with smaller transducer topology), this high-frequency signal evaluation necessitates a uP unit with high computing power and complicated software.

The method proposed in this article utilizes a similar principle of force sensing as in [9], but the evaluation and detection methods of the LC transducer resonant frequency are different, and do not measure the resonant frequency itself. This study uses the identical, described transducer (see Figure 1a), radiator (see Figure 3a), and loading structure (as seen in Figure 1 and Figure 5a,b) as the reflected wave measurement method.

Modifying the measurement method to process the information about applied force at lower frequencies makes hardware and software requirements much lower. This force-sensing method (which may use MEMS structures in millimeter and micrometer ranges) is more accessible for commercial use because it does not require RF components or high-frequency evaluation in orders of GHz, but only in the lower MHz range.

## 3. Electrical Synthesis of Mechanical Structure-Converter (Radiator + Transducer)

The new detection method intends to use a high-frequency signal sweep generated at the voltage-controlled oscillator (VCO) output. This signal wave travels directly towards the radiator port. The ideal (but not required) case occurs when the radiator is a fully closed structure (depending on the resonant frequency of the transducer, the radiator may be a simple directional antenna), with the electromagnetic field being generated only inside such a structure. A mechanical element with a parallel resonance circuit (PRC) creating a transducer (see Figure 1a) is placed within the electromagnetic field produced by the enclosed TEMcell (radiator) (see Figure 3a). Similarly to the previous study, the applied force causes the deformation of the transducer, resulting in a change in the PRC resonant frequency (single component force deformation).

The resonant circuit (PRC) affects the scattered electromagnetic field generated by the radiator. The most significant change occurs at frequencies near the resonant frequency of the converter, where the reflected wave travels back to the output of the VCO and changes the power distribution on the VCO terminal transistors. This power disturbance can be detected on the DC power supply of the VCO. If the statement proves true, high-frequency evaluation to detect the resonant frequency of the transducer can be eliminated.

**Hypothesis 1.**  *The reflected wave influences the VCO, especially the power distribution of the VCO terminal transistors. In the following chapters, we will analyze how this power distribution influences the DC supply current of the VCO to find out if the information about the applied force is measurable.*

First, it is necessary to investigate the interaction between the VCO and the connected load (radiator + transducer), as seen in Figure 3a. The problem with this investigation comes with the division of the system into two separate circuits.

While the VCO is a real electronic circuit that can be simulated in dedicated simulators, the converter (radiator + transducer) appears as a mechanical structure with an unknown electrical equivalent.

The radiator is connected to the VCO directly, which means these two elements affect each other. To quantify this phenomenon, a circuit simulator based on SPICE was used. Both the converter (radiator + transducer) and the VCO has to be represented by their equivalent electrical circuits. The analysis of currents through the VCO terminal transistors and a frequency response of a load impedance (radiator + transducer represents the load impedance) is necessary to obtain the frequency response of the VCO supply current (load of the VCO is frequency-dependent). The frequency response of the current will determine if the change in force applied to the transducer is observable, which is one of the goals of this study.

To find the equivalent circuit of the converter (radiator + transducer), its circuit parameters have to be known. Because it is easily measurable by a network analyzer, measurement of the converter’s input impedance, $Z_{1}$ (see Figure 3a), as a function of frequency appears to be the most logical choice.

The measurement was performed over a frequency range from 100 to 200 MHz by a *Hewlett Packard HP8753D* network analyzer, because this is the frequency range, where the resonance of the converter (radiator + transducer) occurs. The output file contains the frequency-dependent real and imaginary components of the converter input impedance (radiator + transducer).

The impedance response of the converter is shown in Figure 3b which shows a shift of the real impedance maximum value with respect to the middle of the imaginary part overshoot. The impedance response is shown as a function of the frequency sample rather than the frequency itself to simplify further mathematical evaluation. Based on the impedance response, the equivalent circuit consists of multiple resonant circuits, or more specifically, multiple reactive elements.

Let us proceed similarly to designing a topology of a frequency filter. We approximate $Z_{1}\left(f\right)$ by a function:

(1) $$f\left(n\right)=k_{0}+k_{1}s\left(n\right)\frac{a_{0}+a_{1}s\left(n\right)+a_{2}s^{2}\left(n\right)+a_{3}s^{3}\left(n\right)+\cdots }{b_{0}+b_{1}s\left(n\right)+b_{2}s^{2}\left(n\right)+b_{3}s^{3}\left(n\right)+\cdots }$$

where $k_{0},a_{0},\cdots ,a_{N},b_{0},\cdots ,b_{N}$ are desired coefficients. There are 200 samples with first obtaining a value of 100 MHz and the last a value of 200 MHz which gives a number of 200−1 intervals. Then s(n) represents a normalized complex frequency of the respective samples:

(2) $$s\left(n\right)=j+\frac{j}{200}\left(n-1\right)$$

where *n* is the number of samples in a series of values. The *n* was used instead of the frequency *f*. When using frequencies *f* at the orders of $10^{8}$, the convergence could not be achieved by using a function *FindFit* in the computation software *Mathematica*. Subsequently, results have to be calculated to represent the working frequency. Regarding the size of the approximate function and deviations of the results, the best fit came at a function of 4-th order, resulting in:

(3) $$f\left(n\right)=46.24+0.00299\;s\left(n\right)+\frac{151.147-65.43\;s\left(n\right)-74.83\;s^{2}\left(n\right)-35.46\;s^{3}\left(n\right)-62.22\;s^{4}\left(n\right)}{50.74+46.34\;s\left(n\right)+37.23\;s^{2}\left(n\right)+19.08\;s^{3}\left(n\right)+6.42\;s^{4}\left(n\right)}$$

To denormalize the complex frequency, and to preserve the value of s(n), we use:

(4) $$s\left(n\right)\equiv j\omega 10^{-8}\frac{1}{2\pi }$$

Substitution of (4) to (3) results in:

(5) $$\begin{array}{ccc} f(\omega ) & = & 36.55+4.76\cdot 10^{-12}j\omega -\frac{8.06\cdot 10^{7}+2.57\cdot 10^{8}j}{\omega -9.76\cdot 10^{8}-1.19\cdot 10^{7}j}+\frac{8.06\cdot 10^{7}-2.57\cdot 10^{8}j}{\omega +9.76\cdot 10^{8}-1.19\cdot 10^{7}j}\\ & & -\frac{2.35\cdot 10^{9}+7.05\cdot 10^{9}j}{\omega -6.64\cdot 10^{8}-9.21\cdot 10^{8}j}+\frac{2.35\cdot 10^{9}-7.05\cdot 10^{9}j}{\omega +6.64\cdot 10^{8}-9.21\cdot 10^{8}j} \end{array}$$

To visualize the concept, it appears to be advantageous to display the equation in a figure. Each fraction (couple of fractions) in a function (5) is assumed to represent a simple electrical circuit.

**Figure 6:**
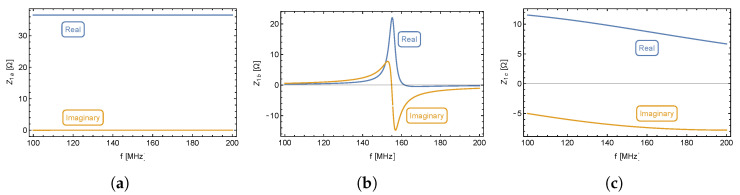
(**a**) First complex number of the Equation (5). (**b**) First and second fraction of the Equation (5). (**c**) Third and fourth fraction of the Equation (5).

**Figure 7:**
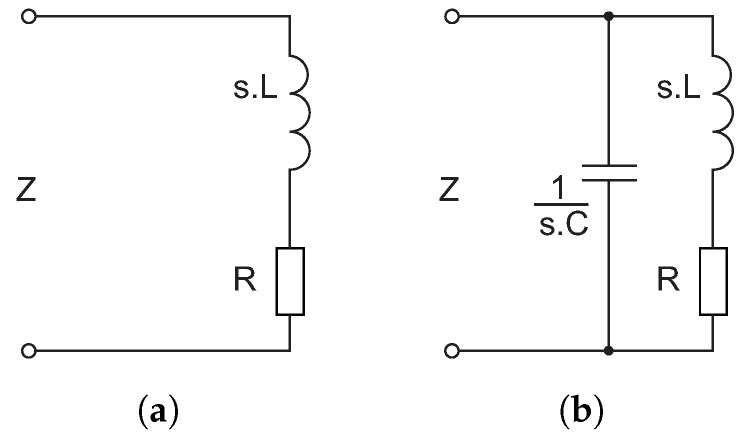
(**a**) Serial RL circuit. (**b**) Parallel resonant circuit.

Figure 6a shows a significant real part of input impedance and a slight positive imaginary part, which appears to be almost constant across the entire frequency band. The circuit representing this figure is a serial RL circuit shown in Figure 7a. Figure 6b clearly documents a resonant circuit and Figure 6c is unambiguous. Its meaning will be clarified in the following analysis.

To synthesize real electronic circuits represented by Equation (5), it appears necessary to start by analyzing the impedance of the parallel resonant circuit (PRC) shown in Figure 7b.

(6) $$Z=\frac{1}{sC+\frac{1}{sL+R}}=\frac{sL+R}{s^{2}CL+sCR+1}=\frac{s\frac{1}{C}+\frac{R}{C\;L}}{s^{2}+s\frac{R}{L}+\frac{1}{C\;L}}$$

where

(7) $$s=\dot{j}\omega$$

Since the first fraction in Equation (5) appears to be similar to the second one, let us try to unify them with the use of a common denominator:

(8) $$\begin{array}{ccc} f_{1,2}\left(\omega \right) & = & -\frac{8.06\cdot 10^{7}+2.57\cdot 10^{8}j}{\omega -9.76\cdot 10^{8}-1.19\cdot 10^{7}j}+\frac{8.06\cdot 10^{7}-2.57\cdot 10^{8}j}{\omega +9.76\cdot 10^{8}-1.19\cdot 10^{7}j}\\ & = & \frac{5.15\cdot 10^{8}j\;\omega +1.63\cdot 10^{17}}{-\omega^{2}+2.39\cdot 10^{7}j\;\omega +9.54\cdot 10^{17}} \end{array}$$

A comparison of (6) and (8) shows clear similarities. The first and second fraction represent the equation of the PRC.

Now, it is possible to calculate the respective PRC elements. Based on Equation (6), the coefficient in the nominator of (8), multiplied by $\dot{j}\;\omega$ , represents the reversed value of capacitance:

(9) $$\frac{1}{C_{1}}=5.15\cdot 10^{8}\;\to \;C_{1}=1.93\cdot 10^{-9}\;\left[F\right]$$

The last coefficient in the denominator of (8) contains elements $C_{1}$ and $L_{1}$ as follows:

(10) $$\frac{1}{C_{1}\;L_{1}}=9.54\cdot 10^{17}\;\to \;L_{1}=5.40\cdot 10^{-10}\;\left[H\right]$$

The last value to calculate is a series resistance for the inductor. By Equation (6), the resistance can be extracted from the second term of the denominator in Equation (8):

(11) $$\frac{R_{1}}{L_{1}}=2.39\cdot 10^{7}\;\to \;R_{1}=0.012\;\left[\Omega \right]$$

The following equation shows the resonant frequency of known elements:

(12) $$f=\frac{1}{2\;\pi \sqrt{C_{1}\;L_{1}}}=1.55481\cdot 10^{8}\;\left[Hz\right]$$

The analyzed PRC represents the real element—the sensing element placed inside the radiator. The sensing element, in regards to its dimensions and function, is an element with continuous parameters and has a significant impact on the shape of the radiator’s input impedance response.

With known elements, this follows the synthesis of the circuit represented by Equation (5):

(13) $$\begin{array}{ccc} f_{3,4}\left(\omega \right) & = & -\frac{2.35\cdot 10^{9}+7.05\cdot 10^{9}j}{\omega -6.64\cdot 10^{8}-9.21\cdot 10^{8}j}+\frac{2.35\cdot 10^{9}-7.05\cdot 10^{9}j}{\omega +6.64\cdot 10^{8}-9.21\cdot 10^{8}j}\\ & = & \frac{1.6131\cdot 10^{19}+1.41122\cdot 10^{10}\dot{j}\omega }{-\omega^{2}+1.84355\cdot 10^{9}j\omega +1.29069\cdot 10^{18}} \end{array}$$

The resulting format of Equation (13) is again similar to (6). It again represents PRC with elements calculated similarly to before. For simplicity and clarity we present only results in the following equations:

(14) $$C_{2}=70.8\;pF,\;\;L_{2}=10.9\;nH,\;\;R_{2}=20.15\;\Omega$$

**Figure 8:**
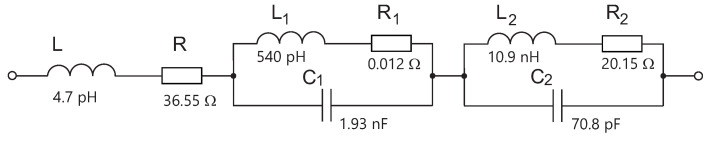
Equivalent circuit of converter (radiator + transducer).

The resonance occurs at a frequency of 180 MHz, while the PRC is significantly attenuated ($R_{2}=20.15\;\Omega$). This circuit incorporates equivalent electric parameters of the radiator and the transducer. The equivalent circuit of high-frequency elements (radiator + transducer), connected to the VCO, consists of an inductor, resistor, and two PRCs as shown in Figure 8.

The answer to the question of this chapter, "What is the VCO electrically connected to?", is now definite: the VCO is loaded by a network of real RLC elements.

## 4. Simulation of VCO Loaded by the Converter (Radiator + Transducer)

**Figure 9:**
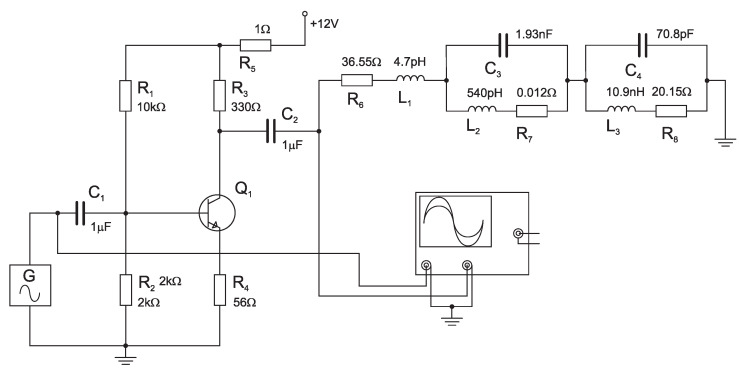
A-class amplifier with load.

**Figure 10:**
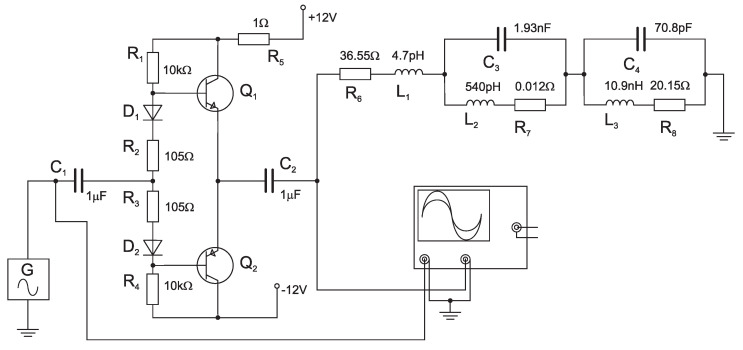
AB-class amplifier with load.

The previous chapter showed the analysis of a high-frequency circuit as a load of VCO and subsequently presented the synthesis of its equivalent circuit as a network of RLC elements. The next step is to simulate and monitor the shape of the VCO DC supply current. The terminal VCO transistors appear to be affected the most. For simplicity, we consider that the VCO has only one final stage connected as a class *A*, or class AB amplifier (see Figure 9 and Figure 10).

In *Multisim* software working on *PSPICE* basis, the final stage/amplifier working in class *A* was modeled, see Figure 9.

**Figure 11:**
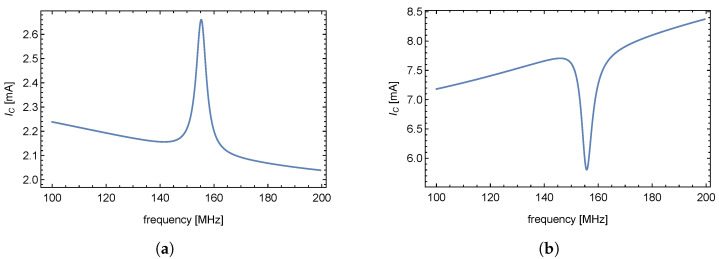
Supply current passing through resistor $R_{5}$ as a function of frequency for A class amplifier (**a**). Supply current passing through resistor $R_{5}$ as a function of frequency for AB class amplifier (**b**).

The circuit consists of *R*, *L*, *C* elements, synthesized in the previous chapter, representing VCO load (radiator + transducer). The signal with constant level 0.2 Vpp was connected at the input (element *G* in Figure 9), while the choice of values of the respective elements takes into consideration the desired amplitude of collector currents. An *AC sweep* module was used to simulate the current passing through resistor $R_{5}$. The resulting figure is a frequency response of collector current via transistor $Q_{1}$ (The current passing through resistor $R_{5}$ is a sum of collector current and the current through resistor divider $R_{1}$ and $R_{2}$ at the base of the transistor. This current is miniscule) shown in Figure 11a. The figure shows the change in the supply current (voltage drop across $R_{5}$) occurring at the resonance frequency of the RLC load. The effect of the transducer resonant frequency on the value of the VCO DC supply current confirms our hypothesis. In this case, the VCO contains a class-*A* amplifier as its final stage.

The study continues by investigating the VCO behavior with the final stage working in class AB, shown in Figure 10. The *AC sweep* module was utilized to simulate the current passing through resistor $R_{5}$ in this case as well.

Similarly to the previous example, the curve of the amplifier supply current (see Figure 11b) shows an overshoot at the resonant frequency of the synthesized converter shown in Figure 8.

In both cases (the difference between the class A and AB amplifier simulation in Figure 11a,b is caused by internal voltage/current sources of the SPICE models and does not reflect the actual current polarity), the influence of converter (radiator + transducer) resonance frequency on the VCO supply current, and therefore the force applied to the transducer, is observable in the shape of the DC supply current. This discovery allows for the modification of the detecting method block diagram shown in Figure 4a, as was intended at the beginning of this study.

The goal of the following chapter will be to eliminate high-frequency components shown in Figure 4a and to focus on detecting the converter resonance in the VCO supply current. Since this goal appears to be achievable, and an overshoot of the VCO supply current seems to carry the information (during the resonance of converter) about the force applied to the transducer, the experimental measurement with the new detection method can be conducted.

## 5. Experimental Setup for the Validation of New Evaluating Method

**Figure 12:**
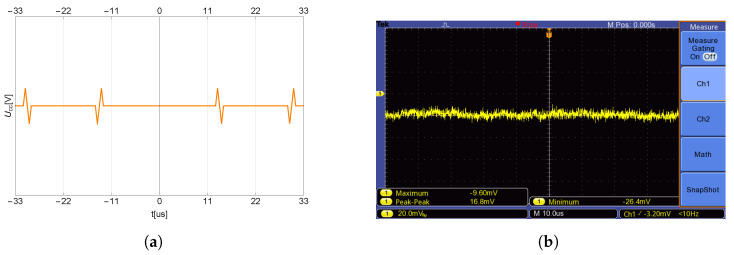
Expected supply current overshoot (**a**) Real supply current overshoot (**b**).

Based on previous chapters, the deformation of a mechanical element (transducer) causes changes in the electrical attributes of the measurement network. A significant influence of the applied force on the transducer is detectable in the overshoot of the VCO supply current. In the case of VCO output frequency being regulated by an input triangle periodic signal, the appearance of periodically repeated ripples of DC supply current is expected, as seen in Figure 12a. Information about physical quantity (force applied to the transducer) is carried by the time delay of consecutive overshoots of VCODC supply current.

The following will focus on the analysis and testing of a reasonable solution for an electronic part of a sensor. This electronic part will be able to reliably detect the overshoot in the VCODC supply current, applying previous hypothetical chapters about our new resonant frequency detection method in a real environment.

The simplest way to measure the VCODC supply current (and subsequently the force applied to the transducer) appears to be to measure the voltage on the current probe. As a VCO, ZX95-200+ by *Minicircuits* was used. Despite the high theoretical overshoot given by analytical and numerical methods in the previous chapter, the overshoot in a real environment may prove difficult to detect. Figure 12b shows that the overshoot of DC supply current can be obscured by background noise (Figure 12b was obtained by an oscilloscope *Tektronix TBS1052B* connected to a 10Ω sensing resistor in the VCO power supply). Due to the fact that the overshoot (information about the physical quantity) was obscured, another solution had to be utilized.

The better (not ideal) solution appears to be to replace the resistor $R_{5}$ (see Figure 9 and Figure 10) with a PRC2 tuned to the frequency of overshoot (if we sweep the control signal of VCO by a triangle signal of 23 kHz, the base frequency of the overshoot is 454 kHz [12]) and again measure the voltage drop. The PRC2 [12] will highlight the overshoot of supply current, which subsequently leads to its easy measurement by available resources.

**Figure 13:**
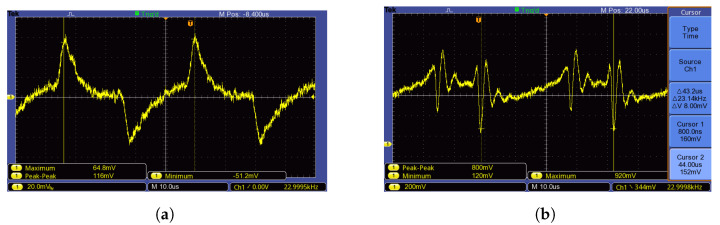
The supply current as a result of voltage drop measurement at PRC2 affected by leaking control voltage (**a**). The same measurement with suppressed crosstalk between control voltage of VCO and supply voltage of VCO (**b**).

Figure 13a shows the time response of the supply current obtained by the voltage measurement on added PRC2 as a current probe. Now, we can see the oscillations of the VCO supply current. The shape, however, is clearly affected by the crosstalk of the leaking VCO triangular control signal. The leaking triangular signal severely complicates the digitization of the resulting signal and its subsequent evaluation by uP. Neither analog nor digital solutions to evaluate the crosstalk-affected signal were proven to be effective, or they required complicated hardware [12] that would negate the simplicity of this solution.

**Figure 14:**
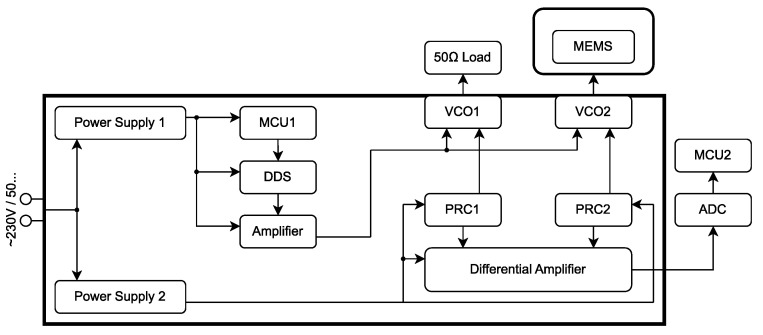
Block diagram of mechanical quantity (single component force) sensing device.

To measure the signal overshoot of the real VCO supply current with minimal crosstalk influence and sufficient information about the applied physical quantity, the electronic circuit shown in Figure 14 was used. Figure 13b shows supply current with suppressed crosstalk (measured according to Figure 12).

The main thought process behind the block diagram in Figure 14 consisted of using a second VCO to subtract the leaking signal—therefore highlighting the desired overshoot. Three main blocks are responsible for creating the periodic triangle signal with desired frequency: microcontroller unit MCU1, a digital synthesizer DDS, and an amplifier. The rise and fall parts of the triangle control signal are as linear as possible. The triangle signal arrives at the control inputs of VCO1 and VCO2 in parallel. Voltage-controlled oscillators create a harmonic voltage of constant amplitude and frequency, proportional to the instantaneous value of the triangle control signal. The load of VCO1 is a 50Ω characteristic impedance and the load of VCO2 is a converter (radiator + transducer). The supply current of VCO1 and VCO2 is supplied by PowerSupply2 and passes through PRC1 and PRC2. PRC1 generates a voltage drop created only by the noisy (crosstalk triangle signal) signal and PRC2 generates a voltage drop created by the noisy signal, as well as the desired signal. These are connected to individual inputs of the DifferentialAmplifier.

Changes in the DC supply currents of VCOs caused by leaking triangle voltage are then subtracted. The output of the differential amplifier carries information about the physical quantity (single-component force)—Figure 13b. The signal at the output of the differential amplifier is further evaluated by an analog–digital converter (ADC) and visualized by MCU2.

## 6. Measurement and Experimental Results

This chapter presents the implementation of a new frequency resonance detection method to determine the transfer function of a given force transducer.

**Figure 15:**
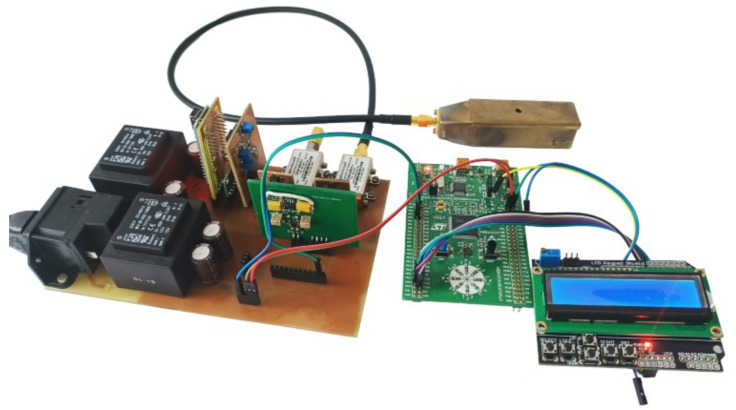
Assembled measurement setup as explained in Figure 14.

The block diagram in Figure 14 was assembled using commercial devices (see Figure 15), while taking into account the linearity of the VCO and the speed of ADC conversion. To test the functionality of the proposed detection method, the microcontroller STM32F3Discovery was chosen as the ADC+MCU2. The best way to sample the signal with the use of ADC is to use DMA (Direct Memory Access) of the controller. By “skipping” the microcontroller processor, it is possible to achieve an ADC sample speed of up to 5MSPS.

**Figure 16:**
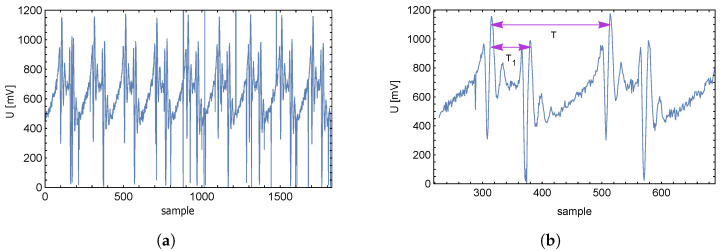
Time (sample) response of supply voltage drop (**a**), and a detail (**b**).

After sampling the signal and saving the sampled signal to memory, the processor of the controller can be used for further signal evaluation. By visualizing the stored data, the response of the VCO supply current as a function of time (individual samples) is shown in Figure 16a.

The level and character of the samples give enough information that it is possible to calculate the time interval between two overshoots of VCODC supply current, and subsequently the force applied to the transducer without the need to know the resonant frequency itself. The computing power of MCU2 is sufficient to compute the intervals $T_{1}$ and *T* on its own. Cycle *T* in Figure 16b is determined by the frequency of the VCO control triangle signal.

The interval $T_{1}$ depends on force applied to the transducer as follows:

(15) $$T_{1}=T-\left(\frac{1}{2\pi \sqrt{\frac{L\varepsilon_{0}\varepsilon_{r}S}{d_{0}-k_{1}F}}}-f_{\min}\right)\left(\frac{T}{f_{\max}-f_{\min}}\right)$$

where $f_{max}=200$ MHz is the maximal VCO output frequency, $f_{min}=100$ MHz is the minimal VCO output frequency, $\epsilon_{0}$ is the vacuum permittivity, $\epsilon_{r}$ is the relative permittivity, *S* is the surface of the capacitor plates, $k_{1}$ is a mechanical constant describing the change in the distance between the plates of the capacitor depending on the force applied to the transducer, $d_{0}$ is the distance between the capacitor plates with no force applied, *L* is the coil inductance of the transducer, and *F* is the applied force to the transducer. A closer analysis is in [10].

(16) $$T_{1}=T\left[2-\frac{\sqrt{d_{0}-k_{1}F}}{k}\right]$$

Equation (15) can be simplified, as shown in Equation (16). Assuming the electrical and mechanical values in Equation (15) are constants, then *k* in Equation (16) is a constant as well (otherwise *k* is a function of force).

The relation between the applied force *F* affecting the transducer and the repeat time (period) of the overshoot $T_{1}$ is nonlinear; however, it is clearly describable by mathematical equations. This fact is necessary to consider while using this method.

**Figure 17:**
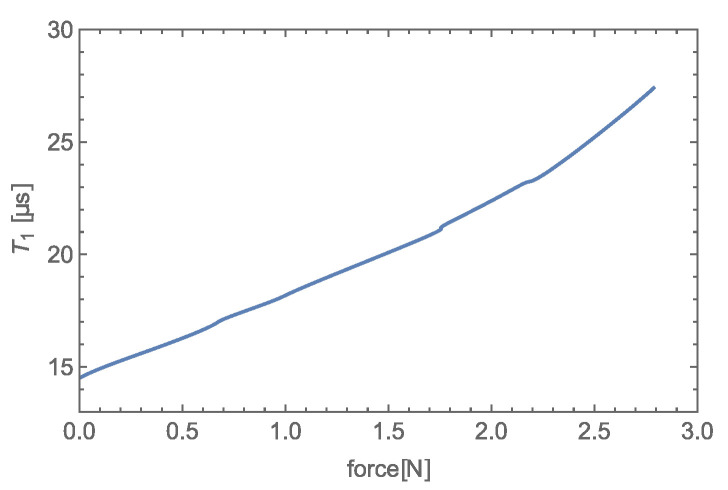
Transfer characteristics of the experimental transducer of a single-component force.

Transfer characteristics based on interval $T_{1}$ (see Figure 17) of given transducer (see Figure 2) were obtained, using single component force similar to that illustrated in Figure 1b.

**Figure 18:**
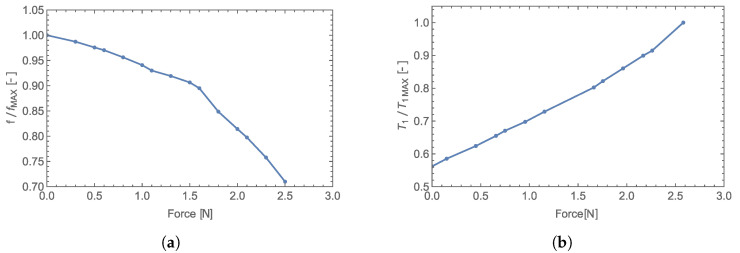
Normalized transfer characteristic (measured with evaluation of reflected wave) (**a**). Normalized transfer characteristic (measured with evaluation of DC supply current of VCO) (**b**).

The same compliant body enclosed in an EM-field-creating radiator was used in the measurement, and the acting force was applied in the same manner as in [9]. The measured transfer characteristic shown in Figure 17 is nonlinear, as expected based on the curve of rising force (see Equation (15)). The normalized transfer characteristics measured by utilizing the VCO DC supply current measurement presented in this study is compared to results in [10] in Figure 18a,b.

As we can see in Figure 4b and Figure 17, the new detection method provides a sensitivity of 4.63 μs/N with a zero offset ∼ 14.5 μs, whereas the old method has a sensitivity of 12.95 MHz/N with a zero offset ∼ 112 MHz with the same transducer.

Interval *T* depends on the sweeping frequency of VCO that is controlled by triangle periodic signal from uP. The measured interval between the VCODC supply current overshoots $T_{1}$ depends on the force applied to the transducer (see Equation (15)). Because the sweeping frequency (frequency of triangle signal) is in the order of tens of kHz, this new resonant frequency detection method needs to measure intervals in the order of μs. The whole measurement can be processed with a cheaper and smaller generic microcontroller, instead of powerful microcomputer (TM4C129X) [9] or network analyzer (when higher frequencies had to be evaluated). Even when MEMS transducers with resonant frequencies in the GHz range are used, the measured interval $T_{1}$ to determine the applied force remains in the μs range (due to the unchanged sweeping frequency).

## 7. Conclusions

The study presents a new force measurement method based on converting force to the PRC resonance frequency and then evaluating the VCO DC current overshoots. This method of detecting resonant frequencies has several advantages. The new method does not require any galvanic connection of a transducer (MEMS) to evaluate electronics, and apart from the other similar measurement methods proposed in [1,2,3,4,5], this method does not need expensive and large equipment to evaluate the information about applied force.

The information transfer about the measured quantity is realized via changes in the electromagnetic field, and the MEMS is not loaded with additional mechanical circuits or devices; therefore, the risk of introducing additional sources of deviation is minimized. The sensitivity and measurement range of force are based on the mechanical structure of the transducer (MEMS) and do not depend on the detection method itself. As a result, this method is applicable to a wide range of force measurement ranges and sensitivities and can be used to detect multiple transducers at once, while the transducers are working on different resonant frequency ranges. While the transducer’s resonant frequency may be in the GHz range, the new evaluation method must process time delays in the µs range, which significantly simplifies the needed hardware and software.

The measurement method has been demonstrated using a scaled version of the MEMS transducer (see Figure 2) to facilitate prototype evaluation.

**Figure 19:**
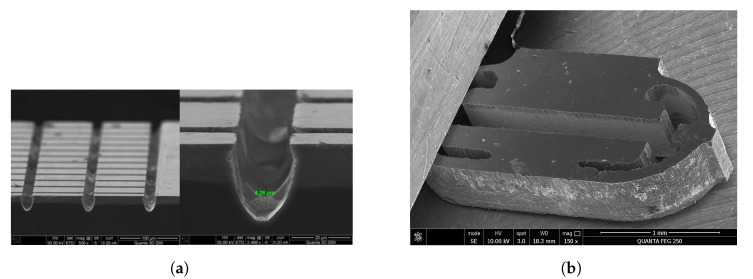
Experimental mechanical element of a transducer in a micrometer range (+ detail) (**a**). Experimental compliant mechanical element of a transducer in a millimeter range (**b**).

As shown in Figure 19b, the mechanical element of the experimental transducer has already been manufactured in the millimeter range. To continue the method assessment with compliant MEMS transducers, manufacturing procedures using reactive-ion etching in the micrometer range have been studied [13], as seen in Figure 19a. These mechanical elements depicted in Figure 19a,b are being investigated further [14,15,16,17,18].

## Data Availability

Data available on request.

## Abbreviations

The following abbreviations are used in this manuscript:

MEMS	Micro electromechanical system
AC	Alternating current
ADC	Analog digital converter
DC	Direct current
DDS	Direct digital synthesis
DMA	Direct memory access
F	Force
MCU	Microcontroller unit
PRC	Parallel resonant circuit
TEM	Transverse electro-magnetic
VCO	Voltage controlled oscillator
VSWR	Voltage standing wave ratio
uP	Microprocessor
EM	Electromagnetic
RF	Radio frequency
